# Morphofunctional Space of the Forelimb in *Caraguatypotherium munozi* (Notoungulata; Mesotheriidae): Insights Into Wrist‐Powered Digging

**DOI:** 10.1002/jmor.70069

**Published:** 2025-07-29

**Authors:** Paul Medina‐González, Karen Moreno

**Affiliations:** ^1^ Departamento de Kinesiología, Facultad de Ciencias de la Salud Universidad Católica del Maule Talca Chile; ^2^ Instituto de Ciencias de la Tierra, Facultad de Ciencias, Laboratorio de Macropaleontología Universidad Austral de Chile Valdivia Chile

**Keywords:** comparative biomechanics, fossorial adaptation, mechanical advantage, miocene mammals, scratch‐digging

## Abstract

Understanding how extinct animals moved is a central goal in paleobiology, yet interpreting locomotor function from anatomy alone is complicated by convergent and divergent morphologies. One promising approach is the construction of morphofunctional spaces (MFSs), which integrate multiple biomechanical indices and comparative statistics to refine functional inference. This study investigates forelimb adaptations for digging in *Caraguatypotherium munozi* (Notoungulata, Mesotheriidae), a mid‐sized Miocene notoungulate lacking extant analogs. We developed an MFS based on osteological measurements and mechanical advantage (MA) models at the elbow and wrist. These were derived from fossil material and comparative data across 38 extant mammal species representing 21 families and 5 locomotor habits—terrestrial, fossorial (digger), climbing, swimming, and flying—as well as 5 mesotheriid specimens, including the holotype of *C. munozi*. Multivariate and inferential statistical analyses were used to identify functional patterns and evaluate locomotor hypotheses. Results show that *C. munozi* occupies an intermediate position in MFS, adjacent to but outside the core regions of extant fossorial, climbing, and terrestrial mammals. It exhibits the highest wrist flexor MA (31.4%) in the data set, 9%–13% above the range of living scratch‐diggers, and a low elbow extensor MA (~19%), below the fossorial mean (~31.6%). Notably, incorporating manus length (MTCIII‐L) into the elbow model lowers MA further but places *C. munozi* within the statistical range of extant diggers, suggesting partial mechanical similarity. This biomechanical pattern supports a wrist‐dominant excavation strategy, reflecting a distinct mechanical pathway that enabled *C. munozi* to perform the motor gesture of scratch‐digging through enhanced distal force generation, rather than relying on proximal joint leverage as in extant fossorial mammals. The integration of MA with osteological indices within a multivariate framework provides novel insights into extinct mammalian locomotion and underscores the utility of MFS models for reconstructing context‐dependent motor capabilities and locomotor habits.

## Graphical abstract

*Caraguatypotherium munozi* displays a distinctive morphofunctional configuration combining high wrist flexor leverage with reduced elbow mechanical advantage—traits consistent with wrist‐dominated scratch‐digging. Multivariate analyses place it outside the core morphospaces of extant locomotor groups. These findings reveal functionally convergent adaptations for excavation among extinct notoungulates.

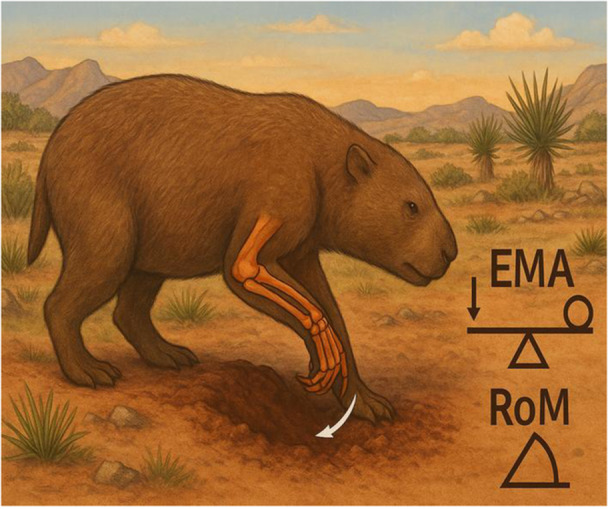

## 1 Introduction

How do mammals move? This fundamental question demands palaeobiological inference grounded in the form–function relationship (Bock and Von Wahlert 1965). Variability in autopodial posture among mammals (Polly 2007), alongside the scaling challenges posed by broad body mass ranges, complicates such inference. In placental mammals, body mass strongly influences autopodial posture: Larger species are generally unguligrade, whereas smaller ones tend to be plantigrade or digitigrade (Kubo et al. 2019; Lovegrove 2004). Although this distribution shows clear trends (Kubo et al. 2019), it is not directly correlated with biological factors such as locomotion type, running speed, or basal metabolic rate (Lovegrove 2004). Furthermore, considerable overlap in locomotor habits exists among plantigrade and digitigrade taxa of small to medium body mass, resulting in overlapping morphospaces (Carrano 1997). This, therefore, can make it difficult to infer locomotor function in extinct mammals. Nevertheless, the movement capabilities of extant mammals—when inferred via morphofunctional analysis—remain poorly understood.

Mesotheriids, a family within Notoungulata, evolved in isolation in South America from the Oligocene to the Late Pliocene, with a distribution spanning Patagonia to equatorial latitudes (Armella et al. 2018; Flynn et al. 2005; Shockey et al. 2007). Their estimated body mass ranges from 22 to 408 kg (Armella et al. 2018; Elissamburu 2012), and they display notable taxonomic and morphological diversity, likely reflecting a broad spectrum of ecological niches (Marshall and Cifelli 1990; Croft 1999). This diversity complicates attempts to infer function or biological capabilities with precision (Shockey et al. 2007; Fernández‐Monescillo et al. 2017). Reports suggest adaptations for swimming (Serres 1867), leaping (Loomis 1914), running (Bond et al. 1995), and digging (Sydow 1988; Shockey et al. 2007).

Interpreting limb function in mesotheriids requires mechanical analogs, as no extant functional homologs exist. Shockey et al. (2007), studying *Trachytherus spegazzinianus* and *Mesotherium cristatum*—representing extremes in mesotheriid body size (Armella et al. 2018)—concluded that all mesotheriids were likely diggers and proposed *Vombatus ursinus* as the closest functional analog. However, apart from work on *Plesiotypotherium achirense* and *M. cristatum* (Fernández‐Monescillo et al. 2017), little palaeobiological research has explored the family more broadly. Under a phylogenetic bracketing approach, all mesotheriids might be assumed to exhibit similar behaviors.

Fernández‐Monescillo et al. (2017) reconstructed forelimb muscle anatomy in mesotheriids using origin and insertion sites to infer force vectors linked to limb retraction, elbow extension, and wrist flexion. However, their model did not assess movement capacity and excluded the autopodium, a crucial element in scratch‐digging kinematics (Moore et al. 2013). This study focuses on *Caraguatyphotherium munozi* from the Miocene of Chile (Flynn et al. 2005; Buldrini et al. 2011), a medium‐sized taxon (Armella et al. 2018) situated centrally within mesotheriid phylogeny (Flynn et al. 2005; Cerdeño et al. 2012; Shockey et al. 2007; Armella et al. 2018; Townsend and Croft 2010), yet poorly known paleobiologically except for a recent paleohistological analysis (Campos‐Medina et al. 2023). Though fossorial ability in mesotheriids has been inferred via functional indices (Fernández‐Monescillo et al. 2017) and digging paradigm tests (Shockey et al. 2007), joint mobility associated with such behavior remains unexplored. The distinction between “function” and “faculty” is often blurred in the literature due to omitted inferential stages, potentially undermining the validity of interpretations (Thomason 1995; Nieto and Rodríguez 2003; Vizcaíno et al. 2016). For instance, the Index of Fossorial Ability (IFA) quantifies the mechanical advantage (MA) at the triceps insertion but does not directly capture biological faculty. It has been demonstrated to apply across multiple locomotor strategies, including both fossorial and aquatic adaptations (Samuels et al. 2013; Chen and Wilson 2015).

Functional indices reveal limb segment movement potential, but do not account for the varied biomechanical strategies behind similar behaviors (Hildebrand and Goslow 2001). To investigate movement comprehensively, it is necessary to assess the full range of possible motor gestures. As Lauder (1995) noted, a single structure can serve multiple functions, and conversely, a function can arise through various anatomical configurations. This phenomenon is formalized in the concept of many‐to‐one mapping of form to function, wherein distinct morphologies can produce equivalent biomechanical outcomes (Wainwright 2005; Wainwright 2007; Losos 2011). These alternative configurations and performance solutions can be systematically explored within morphofunctional spaces (MFS), which accommodate both known and potential functional inferences (Medina‐González and Moreno 2019; Medina‐González 2021a, 2021b; Medina‐González et al. 2022; Vera et al. 2022).

This framework reduces bias by incorporating structured stages for data collection and interpretation (Medina‐González 2021a, 2021b; Vera et al. 2022), ensuring that mechanically relevant traits are meaningfully integrated into functional hypotheses (Gould and Lewontin 1979). Moreover, understanding movement requires attention to the full kinetic chain, particularly how it interacts with substrates to resist linear and angular accelerations—critical in digging behaviors (see Kilbourne and Hoffman 2013).

Digging in mammals encompasses a range of motor strategies, including chisel‐tooth digging (e.g., mole‐rats, gophers), humeral rotation (e.g., moles), scratch‐digging (e.g., armadillos, pangolins, some carnivorans), and hook‐and‐pull digging (e.g., badgers, certain marsupials). These behaviors are often flexible within species and may be combined or alternated depending on ecological context (Hildebrand and Goslow 2001; Nevo 1999; Lacey 2000). Investigating how *C. munozi* may have performed such behavior is facilitated by its well‐preserved forelimb, which includes a nearly complete upper limb, forearm, and manus, with intact metacarpals and phalanges (Flynn et al. 2005; Buldrini et al. 2011).

We focus on a key biomechanical parameter: The MA of the elbow extensors and wrist flexors, which provides a static estimate of force‐generating efficiency based on forelimb lever mechanics (Biewener 1989). This measure is particularly informative for evaluating limb performance in scratch‐digging behaviors, where torque generation and joint‐specific leverage are critical.

By quantifying MA, we aim to characterize the functional morphology of *Caraguatypotherium munozi* and test the hypothesis that it shares greater morphofunctional similarity with extant scratch‐diggers (e.g., armadillos, pangolins, wombats) than with humeral‐rotation diggers (e.g., moles) or non‐fossorial mammals. This hypothesis is evaluated through a multivariate analysis of MA‐based indices, focusing on forelimb traits mechanically associated with scratch‐digging.

As shown in previous studies (Vizcaíno and Milne 2002), biomechanical profiles derived from lever mechanics can effectively distinguish digging strategies by capturing variation in motor patterns and limb use. We propose that if *C. munozi* groups with extant scratch‐diggers in MFS, this would support the hypothesis of wrist‐powered digging through structural similarity. Conversely, a distinct position in morphospace would suggest a different functional adaptation.

Accordingly, we ask: What are the functional ranges of MA in the forelimb of *C. munozi*, and how do these relate to its potential for scratch‐digging? To address this question, we apply a morphofunctional framework grounded in biomechanical indicators of force transmission, enabling a quantitative assessment of structural and functional similarity with extant fossorial mammals.

## 2 Materials and Methods

### 2.1 Material

**Figure 1:**
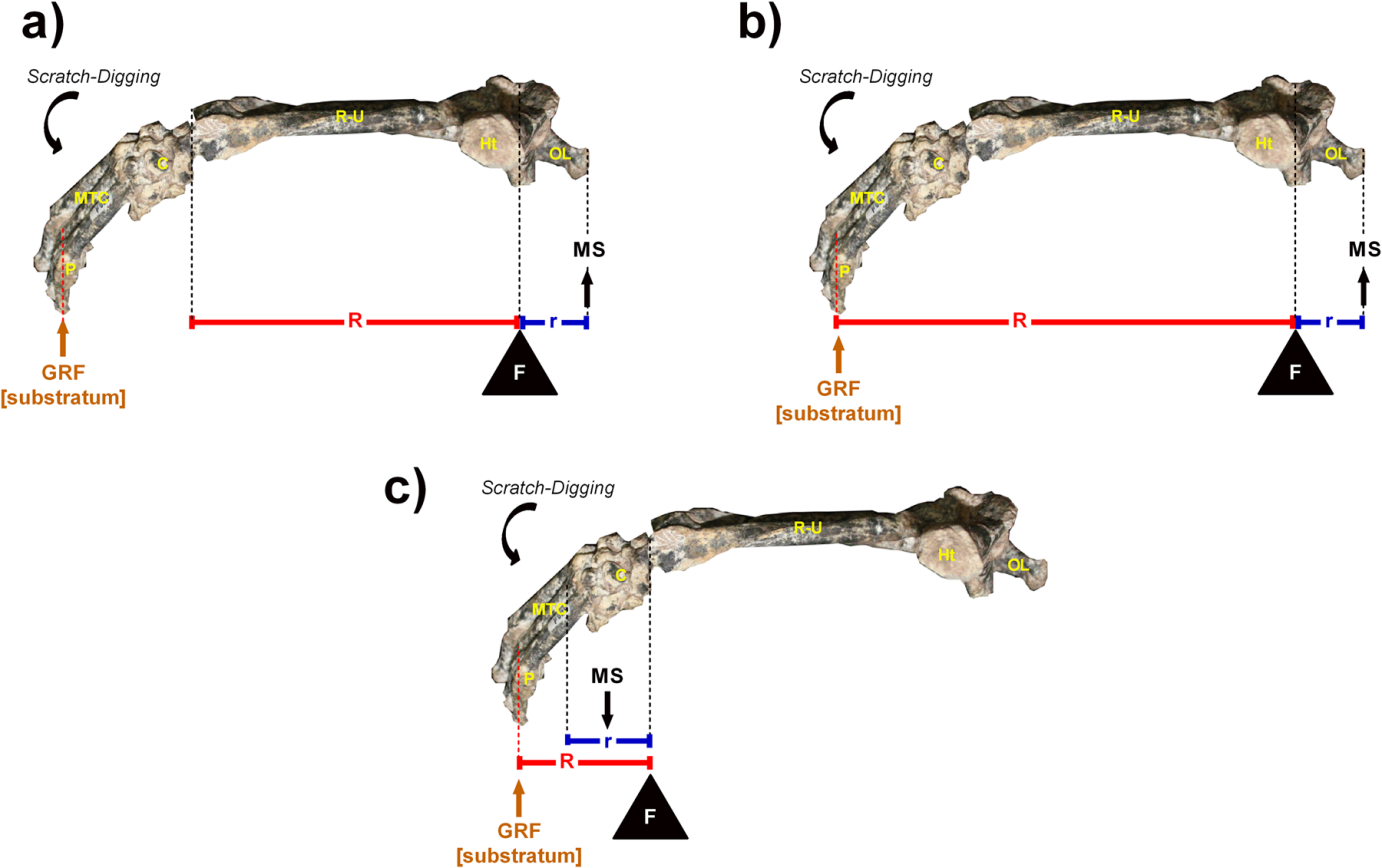
Models of mechanical advantage (MA) applied to the holotype of *Caraguatypotherium munozi* (SGO.PV.22500, MNHN). (a) Classical elbow mechanical advantage model (MAe), equivalent to the Index of Fossorial Ability (IFA), following Hildebrand (1985). (b) Modified elbow model incorporating distal segments—specifically, the third metacarpal and phalanges (MAe + MTCIII‐L). (c) Wrist mechanical advantage model (MAw), with the rotational axis at the carpus. In all models, lever arms were measured as straight‐line distances: resistance arms (R) extended from the joint axis to the distal tip of the ulna (MAe) or to the estimated substrate contact point, approximated by the apex of metacarpal III (MAe + MTCIII‐L and MAw, see Figure 2d). Force arms (r) were defined from the joint axis to the olecranon tip (elbow extensors) or to the proximal base of metacarpal III (wrist flexors). These measurements assume a neutral wrist position and do not account for wrist angulation or sesamoid contributions (e.g., pisiform), and should be interpreted as static osteological approximations. C, carpus; F, fulcrum; GRF, ground reaction force; Ht, humerus transverse; MS, muscle strength; MTC, metacarpus; OL, olecranon; P, phalangesr, force arm; R, resistance arm; R–U, radius and ulna.

We examined the holotype of *C. munozi* (SGO.PV.22500 MNHN), which comprises an incomplete left forelimb. The proximal epiphysis of the humerus is absent, though the remainder of the bone is preserved. The complete forearm includes both the ulna and radius, well preserved and articulated with the distal humerus. The manus retains all metacarpals and the proximal phalanges of digits II, III, and IV. Six semilunar‐shaped sesamoid bones are associated with the metacarpophalangeal joints (Flynn et al. 2005) (Figure 1).

We also obtained measurements from published images of four other notoungulate species across three mesotheriid genera: (i) *T. spegazzinianus* (Shockey et al. 2007), (ii) *P. casirense* (Cerdeño et al. 2012), (iii) *P. achírense*, and (iv) *M. cristatum* (Fernández‐Monescillo et al. 2017) (Table 1).

**Table 1:** Species and their locomotor categorizations.

Locomotor habit	Order	Family	Species
Mesotheriidae fossil record (n, 5)	Notoungulata	Mesotheriidae	*Caraguatypotherium munozi* MNHN‐SGO PV.22500
	Notoungulata	Mesotheriidae	*Plesiotypotherium casirense* MNHN‐Bol‐V 003724
	Notoungulata	Mesotheriidae	*Trachytherus spegazzinianus* UF 91933
	Notoungulata	Mesotheriidae	*Plesiotypotherium achirense* MNHN ACH 18; MNHN‐Bol‐V 12687; MNHN‐Bol‐V 12768; MNHN‐Bol‐V 12768; MNHN‐Bol‐V 12760
	Notoungulata	Mesotheriidae	*Mesotherium cristatum* MNHN‐PAM 2
Digger (n, 4)	Cingulata	Dasypodidae	*Dasypus novemcinctus*
	Rodentia	Ctenomyidae	*Ctenomys maulinus*
	Rodentia	Caviidae	*Cavia porcellus*
	Pholidota	Manidae	*Smutsia gigantea*
Terrestrial (n, 15)	Lagomorpha	Leporidae	*Lepus europaeus*
	Artiodactyla	Bovidae	*Bos taurus*
	Perissodactyla	Equidae	*Equus ferus caballus*
	Carnivora	Canidae	*Canis lupus familiaris*
	Primates	Hominidae	*Homo sapiens*
	Artiodactyla	Cervidae	*Pudu puda*
	Rodentia	Muridae	*Rattus norvegicus*
	Carnivora	Canidae	*Canis lupus familiaris*
	Carnivora	Felidae	*Felis silvestris catus*
	Artiodactyla	Suidae	*Sus scrofa domestica*
	Artiodactyla	Bovidae	*Eudorcas thomsonii*
	Artiodactyla	Bovidae	*Ovis orientalis aries*
	Carnivora	Felidae	*Puma concolor*
	Carnivora	Mustelidae	*Galictis cuja*
	Carnivora	Mustelidae	*Lyncodon patagonicus*
Climber (n, 9)	Microbiotheria	Microbiotheriidae	*Dromiciops gliroides*
	Primates	Cebidae	*Saimiri sp*.
	Primates	Pitheciidae	*Pithecia pithecia*
	Primates	Pitheciidae	*Chiropotes satanas*
	Pilosa	Megalonychidae	*Choloepus didactylus*
	Pilosa	Bradypodidae	*Bradypus variegatus*
	Carnivora	Mustelidae	*Eira barbara*
	Pholidota	Manidae	*Manis pentadactyla*
	Pholidota	Manidae	*Phataginus tricuspis*
Swimmer (n, 8)	Rodentia	Myocastoridae	*Myocastor coypus*
	Carnivora	Mustelidae	*Neovison vison*
	Carnivora	Phocidae	*Hydrurga leptonyx*
	Carnivora	Mustelidae	*Neovison vison*
	Carnivora	Mustelidae	*Mustela putorius furo*
	Carnivora	Otariidae	*Zalophus californianus*
	Carnivora	Mustelidae	*Lontra longicaudis*
	Carnivora	Mustelidae	*Lontra provocax*
Flying (n, 2)	Chiroptera	Molossidae	*Tadarida brasiliensis*
	Chiroptera	Pteropodidae	*Rousettus aegyptiacus*

*Note:* Taxa are grouped by locomotor habit inferred from ecological literature, functional morphology, and skeletal indicators of movement. The five locomotor categories are: Digger—fossorial or semifossorial species adapted for scratch‐digging; Terrestrial—ground‐dwelling quadrupeds with generalized locomotion; Climber—arboreal or scansorial taxa with grasping or climbing adaptations; Swimmer—semi‐aquatic species with swimming specializations; Flyer—volant mammals using forelimb‐derived wings. Locomotor assignments for fossil taxa (e.g., *Caraguatypotherium munozi*) are based on skeletal proportions and comparative context. Full specimen information, measurements, and data sources are provided in File S1.

In addition, we included 38 extant mammalian specimens from 21 families, categorized by locomotor habit: diggers (*n* = 4), terrestrials (*n* = 15), climbers (*n* = 9), swimmers (*n* = 8), and flyers (*n* = 2) (Chen and Wilson 2015; Meachen‐Samuels and Van Valkenburgh 2009; Samuels et al. 2013; Shimer 1903) (Table 1). Specimens were examined and measured between 2017 and 2019 at the Comparative Anatomy Laboratory and the Collections Section of the Faculty of Veterinary Sciences, Universidad Austral de Chile. Sample sizes for modern taxa were selected to maximize taxonomic and functional diversity across locomotor strategies, following standards used in previous comparative biomechanical studies (e.g., Chen and Wilson 2015; Samuels et al. 2013). Further measurements were obtained from scaled images in the literature showing forelimbs in cranial and caudal views (English 1977; Ercoli 2015; Fleagle and Meldrum 1988; Gaudin et al. 2016; Nyakatura and Fischer 2011; Olson et al. 2016). Additional details are provided in File S1.

### 2.2 Osteological Measurements and Functional Indices

**Figure 2:**
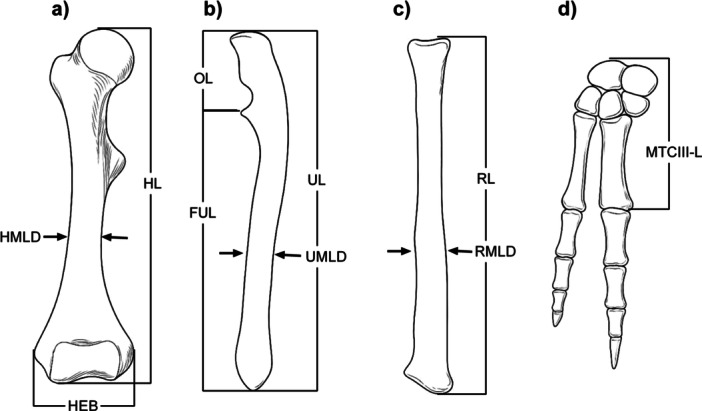
Linear osteological measurements used for calculating functional indices and mechanical advantage models in the forelimb. Schematic lateral view of a generalized mammalian forelimb showing: (a) Humerus—total length (HL), midshaft lateral diameter (HMLD), and epicondylar breadth (HEB). (b) Ulna—total length (UL), functional length excluding the olecranon (FUL), midshaft lateral diameter (UMLD), and olecranon length (OL). (c) Radius—total length (RL) and midshaft diameter (RMLD). (d) Manus—including the carpus and third metacarpal (MTC III). MTCIII–L is defined as the linear distance from the wrist joint (carpal‐metacarpal articulation) to the distal tip of MTC III, and was used to estimate the resistance lever arm in the modified elbow and wrist mechanical advantage models. The force lever arm for wrist flexors was measured from the wrist joint to the proximal base of MTC III, approximating the insertion point of tendons such as *flexor carpi radialis* and *flexor carpi ulnaris*; this is schematized in Figure 1c. All linear measurements were recorded in centimeters and log‐transformed for body size correction using regressions against humeral length. Functional indices and mechanical advantage ratios were calculated using the formulas in Table 2. Measurement definitions follow Elissamburu (2013), with modifications to MTCIII–L from Samuels et al. (2013), and MA models based on the conceptual framework of Biewener (1989).

Osteological measurements and functional indices were obtained following established methodologies (Chen and Wilson 2015; Elissamburu 2013; Fernández‐Monescillo et al. 2017). Table 2 and Figure 2 outline the variables and formulae used in this study.

**Table 2:** Description of osteological measurements, functional indices, and mechanical advantage (MA) models used in this study.

Osteological measurements	Ab.	Definition
Humerus length	HL	Length between the extremities of humerus.
Humerus mid‐lateral diameter	HMLD	Largest diameter of the humerus shaft.
Humerus epicondylar breadth	HEB	Diameter between the condyles of the humerus.
Ulna length	UL	Length between the ends of the ulna.
Functional ulna length	FUL	Length between the ends of ulna minus olecranon.
Ulna mid‐lateral diameter	UMLD	Largest diameter of the ulna shaft.
Olecranon length	OL	Length from the elbow center of rotation to the end of the olecranon.
Radius length	RL	Length between the extremities of radius.
Radius mid‐lateral diameter	RMLD	Largest diameter of the radius shaft.
Manus through Carpal–Metacarpal III Length	MTCIII‐L	Linear distance from the wrist joint line to the distal end of the third metacarpal (MTC III).

Functional indices	**Ab.**	**Calculation**
Humerus robustness index	HRI	[HMLD/HL] × 100
Epicondylar index	EI	[HEB/HL] × 100
Index of fossorial ability	IFA	[OL/FUL] × 100
Olecranon index	OI	[OL/UL] × 100
Brachial index of radius	BIR	[RL/HL] × 100
Brachial index of ulna	BIU	[UL/HL] × 100
Radial robustness index	RRI	[RMLD/RL] × 100
Ulna robustness index	URI	[UMLD/UL] × 100

**MA**	**Ab.**	**Calculation**
MA at elbow (original model)	MAe	[r (elbow extensors)/R(FUL)] × 100
MA at elbow (+metacarpus)	MAe+ MTCIII‐L	[r (elbow extensors)/R(FUL + MTCIII‐L)] × 100
MA at wrist	MAw	[r (wrist flexors)/R(MTCIII‐L)] × 100

*Note:* Measurements and indices follow definitions from Elissamburu (2013). MTCIII‐L was modified from Samuels et al. (2013) to represent the functional length from the wrist joint line to the proximal base of the third metacarpal, rather than the anatomical length of the isolated bone and mechanical advantage (MA) based on the framework originally proposed by Biewener (1989). All linear measurements are in centimeters. Abbreviations and definitions for each variable are provided on the table. BIR and BIU represent stylopod–zeugopod proportions, with BIR reflecting reach potential and forearm curvature (Fujiwara and Hutchinson 2012), and BIU reflecting distal torque and triceps leverage (Kardong 2012; Kilbourne and Hutchinson 2019; Hildebrand 1985). All measurements are in cm. See File S1 for a full data set. For *Caraguatypotherium munozi* (SGO.PV.22500), humeral length was estimated based on ulna/humerus proportions derived from a conspecific adult specimen preserving both elements (Campos‐Medina et al. 2023).

Depending on specimen availability, two procedures were applied. Direct measurements were taken from dry skeletons using a Uyustool CLA006 caliper (150 mm, 0.02 mm precision). Alternatively, photogrammetric analyses were performed on scaled images using Tracker 4.11.0 (Brown 2022). This protocol demonstrates excellent inter‐rater reliability, with an Intraclass Correlation Coefficient of 0.98 (95% CI = 0.96–0.99, *p* < 0.001), and strong criterion validity (Pearson's *r* = 0.96; 95% CI = 0.92–0.98; *p* < 0.001) relative to expert measurements (Medina‐González 2014; Medina‐González 2021a; Vera et al. 2022), see File S2.

As the proximal epiphysis of the humerus was missing in the holotype of *C. munozi* (SGO.PV.22500), we estimated total humeral length for proportional index calculations using an integrative approach based on conspecific allometry. Specifically, we applied the ulna/humerus ratio observed in another adult specimen of *C. munozi* (GEOUACH.HS.HD.1), which preserves both elements and yields a ratio of 1.19 (ulna = 23.2 cm; humerus = 19.53 cm; Campos‐Medina et al. 2023). Given that the ulna length (UL) in the holotype is 16.26 cm, the estimated humerus length (HL) is 13.66 cm. This inferred value was used solely for index calculations (i.e., HRI, EI, BIH, BIU) and is explicitly flagged in the data set.

### 2.3 Mechanical Advantage: Elbow and Wrist Models

Lever mechanics were employed to estimate the MA of muscle groups during scratch‐digging movements (Biewener 1989). However, following Biewener (1989) definition, we acknowledge that “effective mechanical advantage” (EMA) refers specifically to dynamic conditions involving ground reaction force vectors and joint angles during movement. As our models are based on fixed osteological measurements, we use the term “mechanical advantage” (MA) to reflect this static approach.

We developed three MA models that considered: (i) the location of the joint axis (elbow or wrist); (ii) the force lever arm; and (iii) the resistance lever arm (Figure 1).

The first model represents a classical elbow‐centered rotation system (first‐class lever), also known as the IFA (Hildebrand 1985) or Olecranon Process Length Index (Sargis 2002), Figure 1a. The second model also centers the joint axis at the elbow, but incorporates the length of the manus (Elissamburu 2007; Rose et al. 2014), Figure 1b. The third model follows a distal‐to‐proximal recruitment pattern described in wombats—considered functional analogs of mesotheriids (Shockey et al. 2007)—and positions the joint axis at the wrist, representing a third‐class lever system (Figure 1c).

Resistance lever arm length was measured from the joint axis (elbow or wrist) to the distal end of the ulna in the classical model. In the modified elbow and wrist models, resistance was measured from the joint axis to the estimated substrate contact point, which was approximated by the distal tip of the third metacarpal (MTCIII‐L) as defined in Table 2 and illustrated in Figure 2d. This distal tip was used as a proxy for the point of ground contact during scratch‐digging.

Muscle force lever arms were defined as the linear distance from the joint axis to the apex of the olecranon (for elbow extensors, primarily *triceps brachii*) and to the proximal base of metacarpal III (for wrist flexors, including *flexor carpi radialis* and *flexor carpi ulnaris*). It is important to emphasize that estimating wrist flexor moment arms as a straight‐line distance from the wrist joint to the base of the third metacarpal constitutes a simplification of the actual anatomical configuration (Figure 1c). In vivo, flexor tendons typically run parallel to the long axis of the antebrachium and manus, and the effective moment arm is more directly influenced by the dorsoventral thickness of the proximal manus. This is particularly relevant for the *flexor carpi ulnaris*, whose mechanical leverage is enhanced by the presence of the pisiform—a prominent sesamoid bone that is not fully represented in our model. Therefore, the values presented here should be considered static osteological approximations, rather than exact reconstructions of dynamic tendon mechanics.

The corresponding calculation equations are listed in Table 2, with species‐specific data provided in File S1.

### 2.4 Statistical Analysis

Descriptive statistics for osteological measurements, functional indices, and MA values are reported as means ± one standard deviation. To evaluate differences among extant locomotor groups, one‐way analyses of variance (ANOVA) were conducted separately for each variable, followed by Tukey's Honestly Significant Difference tests for post hoc pairwise comparisons when significant main effects were detected. Superscript letters (a, b) in Table 3 indicate statistically homogeneous groups for variables with significant ANOVA results.

**Table 3:** Functional indices and MA values for modern and extinct mammals (Mesotheriidae family) grouped by locomotor habit.

Variables [%]	Locomotor habits (extant mammals)					Mesotheriidae fossil record	
	Digger [*n* = 4]	Terrestrial [*n* = 15]	Climber [*n* = 9]	Swimmer [*n* = 8]	Flyer [*n* = 2]	Other mesotherids [*n* = 4]	*C. munozi* [*n* = 1]
HRI	12.5 ± 5.1	9.7 ± 3.3	10.3 ± 5.6	12.6 ± 6.2	5.1 ± 0.2	15.5 ± 3.3^e^, ^h^	11.57^e^, ^h^
EI	33.9 ± 11.5^a^	21.9 ± 4.4^b^	27.3 ± 11.3^a,b^	31.2 ± 6.4^a^	10.5 ± 0.8^c^	33.9 ± 3.6^e^, ^h^	30.01^e^, ^h^
OI	22.9 ± 9.9^a^	18.1 ± 7.6^a^	13.7 ± 8.5^a^	21.4 ± 2.3^a^	2.8 ± 3.6^b^	21.2 ± 8.2^h^	16.30^h^
BIR	75.7 ± 16.7^a^	87.1 ± 16.8^a^	91.0 ± 20.0^a^	75.7 ± 15.4^a^	171.5 ± 1.6^b^	88.8 ± 7.3^h^	89.97^h^
BIU	110.6 ± 16.5	107.1 ± 17.4	104.2 ± 18.2	95.7 ± 14.3	126.1 ± 62.5	119.0 ± 11.0^g^	119.03
RRI	12.9 ± 8.0	10.0 ± 3.7	8.5 ± 7.2	10.6 ± 4.2	3.2 ± 0.2	8.7 ± 1.1^h^	7.00^h^
URI	12.4 ± 7.6	6.8 ± 3.7	9.6 ± 7.3	11.0 ± 4.2	3.4 ± 0.4	13.0 ± 6.1^h^	8.30^h^
MAe (IFA)	31.6 ± 19.1	23.2 ± 12.3	17.1 ± 14.0	27.3 ± 3.8	2.9 ± 3.9	27.9 ± 12.2^h^	19.47^h^
MAe+ MTCIII‐L	16.0 ± 8.3^a^	11.1 ± 4.8^a,b^	9.2 ± 5.5^a,b^	12.8 ± 2.7^a,b^	0.8 ± 1.1^b^	14.8 ± 6.3^h^	12.51^h^
Maw	18.4 ± 5.8	17.5 ± 5.5	20.5 ± 7.8	18.8 ± 5.1	6.3 ± 0.8	27.7 ± 2.3^d^, ^e^, ^f^, ^g^, ^h^	31.43^d^, ^e^, ^f^, ^h^, ^i^

*Note:* Values are presented as mean ± standard deviation. For *Caraguatypotherium munozi*, values correspond to the holotype (MNHN, SGO.PV.22500), with humeral length estimated from anatomical reconstructions (Campos‐Medina et al. 2023). Data for other mesotheriids include *Trachytherus spegazzinianus*, *Plesiotypotherium casirense*, *P. achírense*, and *Mesotherium cristatum*. IFA corresponds to the conventional mechanical advantage of the elbow joint. Superscript letters (a, b) denote statistically significant differences among extant locomotor groups, based on one‐way ANOVA followed by Tukey's HSD test (*p* < 0.05). Groups sharing the same letter do not differ significantly. Letters are shown only for variables with significant ANOVA results (EI, BIR, OI, MAe + MTCIII‐L). Variables without significant group effects (HRI, URI, BIU, RRI, MAe, MAw) are not annotated with letters. Superscript letters (c–h) indicate significant differences (*p* < 0.05) between fossil values and specific extant groups. Letters next to *C. munozi* values reflect one‐sample *t*‐tests; letters next to other mesotheriid values reflect Welch's *t*‐tests: Letters are only shown for statistically significant comparisons. All indices are expressed as percentages (%).

Abbreviations: e, elbow; MA, mechanical advantage; MAe +  MTCIII‐L, combined elbow MA and metacarpal III length; w, wrist.

^d^ Significantly different from diggers.

^e^ Terrestrials.

^f^ Climbers.

^g^ Swimmers.

^h^ Flyers.

^i^ Other mesotheriids.

To assess the functional distinctiveness of *C. munozi*, one‐sample *t*‐tests were used to compare its values with each extant locomotor group. In addition, independent samples *t*‐tests (using Welch's correction) were performed to compare the mesotheriid group against each modern locomotor category.

Principal component analysis (PCA) was used to evaluate interspecific variation in forelimb morphology. A total of 10 log‐transformed osteological variables and 7 functional variables, including four percentage‐based limb indices and 3 MA models, were included in the analysis.

To reduce the effect of body size on linear osteological variables, we applied a size correction based on log–log regression residuals. Each variable was log‐transformed and regressed against log‐transformed HL, which served as a proxy for overall limb size. The resulting residuals, representing size‐independent morphological variation, were then standardized (*z*‐transformed) and used as input for PCA. This procedure emphasizes shape‐ and proportion‐based differences in skeletal morphology, minimizing the confounding effects of absolute size (File S1).

Statistical significance was set at *p* < 0.05. All statistical analyses and figures were generated in GraphPad Prism 6.0 (2012), and PCA was conducted in RStudio (RStudio Team 2020), with PCA visualizations assisted by ChatGPT to complement R‐based outputs. All procedures followed Chilean regulations (Letter No. 1355/2023). Data and R scripts are provided as Supporting Information Files and archived in Zenodo (DOI: 10.5281/zenodo.15189182).

## 3 Results

### 3.1 Osteological Measurements of Forelimbs

**Figure 3:**
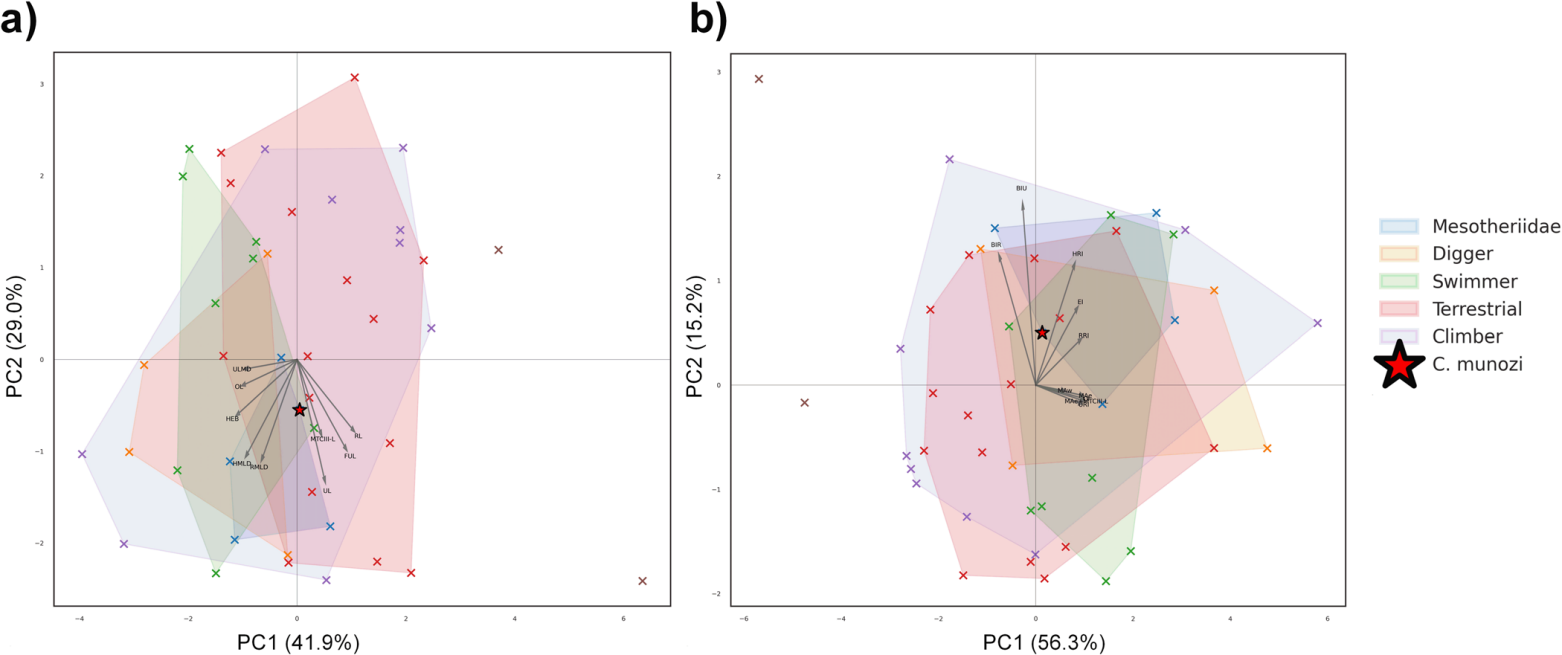
Principal component analysis (PCA) of morphofunctional variation in mammalian forelimbs. (a) Morphospace based on nine osteological variables, log‐transformed and size‐corrected by regression against log‐transformed humerus length. Residuals were *z*‐standardized to emphasize shape over size. PC1 and PC2 explain 41.9% and 29.0% of the total variance, respectively. (b) Morphospace derived from 10 functional traits, including seven limb indices and three mechanical advantage (MA) metrics. PC1 and PC2 account for 56.3% and 15.2% of the variance, respectively. Convex hulls delineate the distribution of extant taxa according to locomotor habit. Arrows indicate principal component loadings: their direction shows the axis of trait increase, and their length reflects the strength of correlation with each component. *Caraguatypotherium munozi* is marked by a red star with a black outline. Its position across both spaces reflects unique combinations of forelimb proportions and mechanical traits compared to modern analogs. Full measurement data and taxonomic information are available in File S3.

PCA of nine log‐transformed, size‐corrected osteological variables yielded two principal axes that together explain 70.85% of the total variance (PC1: 41.9%; PC2: 29.0%; Figure 3a; Table S3.1).

PC1 primarily captures variation in limb segment proportions, with high positive loadings from radial length and functional UL, and negative loadings from midshaft diameters (e.g., HMLD, RMLD, ULMD), reflecting a shape gradient from gracile to robust limbs. PC2 is mainly structured by distal lever‐arm traits, particularly olecranon length (OL), UL, and epicondylar breadth (HEB), which exhibit moderate‐to‐strong negative correlations. These loading patterns are visualized in Figure 3a, where direction and magnitude reflect the contribution of each variable to the PCA structure.

Within this morphospace, mesotheriid taxa form a compact cluster distinct from extant fossorial, terrestrial, or climbing mammals. *C. munozi* plots in an intermediate region of morphospace, driven by intermediate values in humeral and ulnar dimensions, particularly UL and HMLD. These traits place it closer to generalized terrestrial or climbing taxa rather than to specialized fossorial forms, suggesting that *C. munozi* retained a powerful yet generalized forelimb architecture, decoupled from typical scratch‐digger morphotypes.

### 3.2 Forelimb Functional Indices

**Figure 4:**
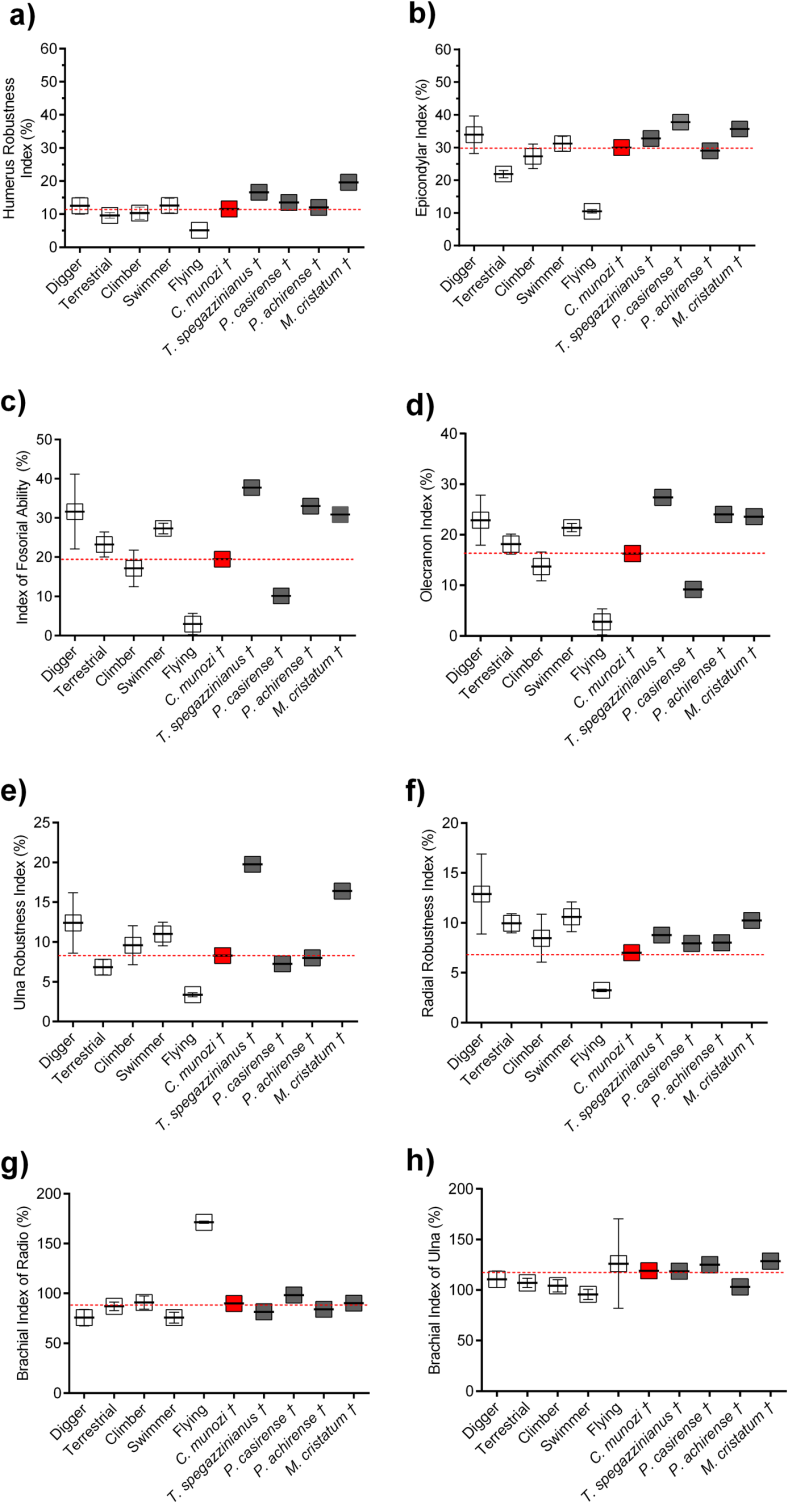
Forelimb functional indices for extant mammals with known locomotor habits (mean ± standard error shown in white) and members of the Mesotheriidae family (dark gray). *Caraguatypotherium munozi* is represented in red. Definitions of all indices are provided in Table 2.

Functional indices derived from linear morphometrics are summarized in Table 3 and illustrated in Figure 3b. *C. munozi* exhibits moderately elevated values for the brachial indices of the radius and ulna (BIR and BIU; Figure 4g–h), consistent with increased leverage in proximal limb segments. These values partially overlap with those of extant fossorial and climbing mammals, although *C. munozi* is differentiated by its unique combination of intermediate brachial ratios and disproportionately high wrist mechanics.

It is important to note, however, that similar index values can result from different anatomical configurations. For example, a high brachial index may arise from humeral elongation or forearm shortening, each implying distinct functional strategies. Such convergences in ratio‐based metrics must be interpreted cautiously and in combination with direct biomechanical evidence.

A PCA of seven functional indices and three MAs values yielded two components that jointly explain 71.48% of the total variance (PC1: 56.25%; PC2: 15.23%; Figure 3b; Table S3.2). PC1 is largely driven by MA variables (i.e., elbow MA “Mae,” wrist MA “Maw,” and the combined elbow–metacarpal III model “MAe+MTCIII‐L”), which exhibit the strongest positive loadings (*r*  ≥  0.365). PC2 is structured by brachial morphology, with BIR (*r*  =  0.478) and BIU (*r*  =  0.672) showing the highest contributions to this axis.

**Figure 5:**
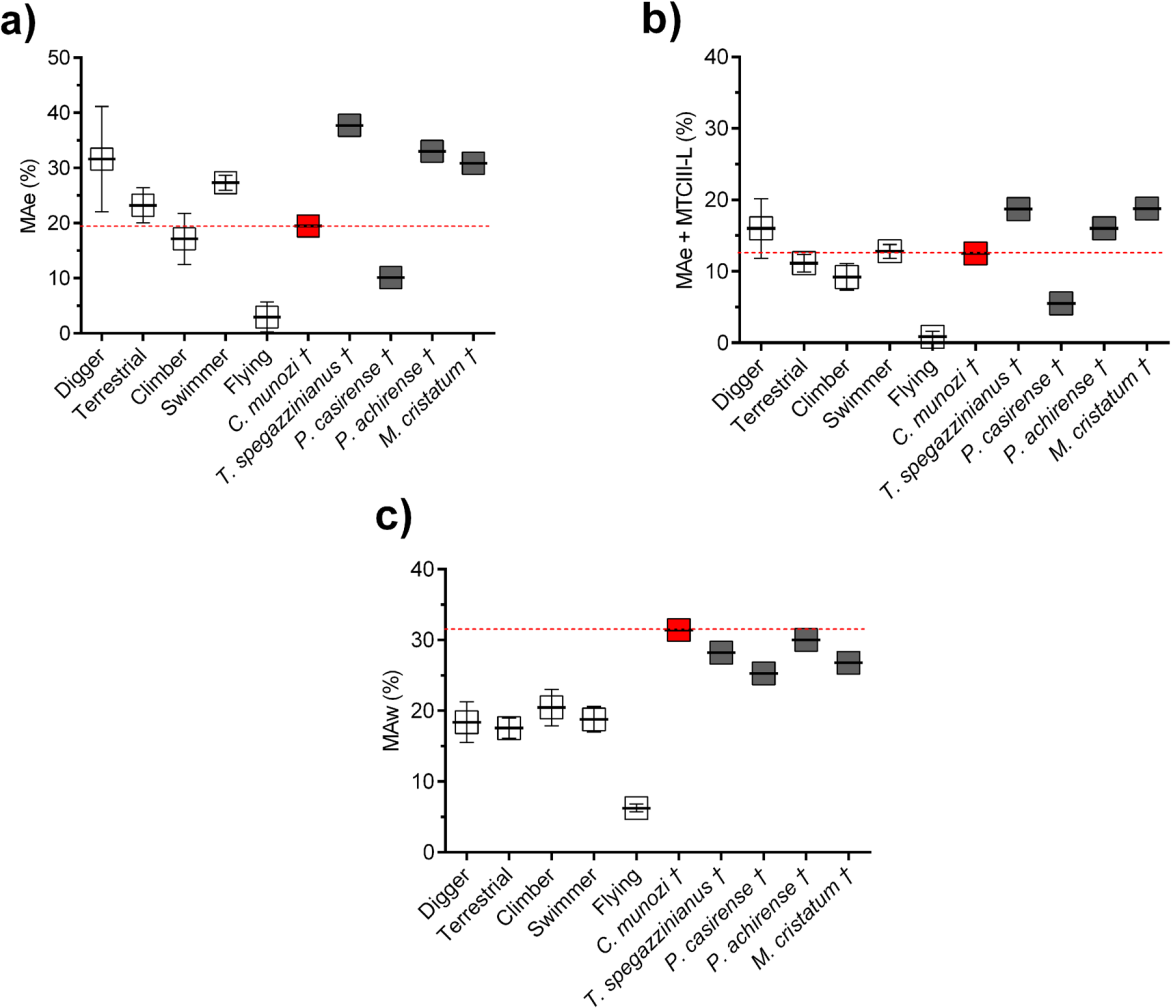
Results of mechanical advantage (MA) models in extant mammals with known locomotor habits (mean ± standard error shown in white) and members of the Mesotheriidae family (dark gray). *Caraguatypotherium munozi* is represented in red. (a) Classic elbow mechanical advantage (MAe), based on the model proposed by Hildebrand (1985); this metric corresponds to the fossorial index illustrated in Figure 3c, reflecting variation in elbow joint leverage across groups. (b) Modified elbow MAe incorporating the contribution of the manus segment (metacarpus and phalanges). (c) Wrist mechanical advantage (MAw), introduced in the present study. Definitions of all EMA models are provided in Figure 1 and Table 2.

In this PCA space, *C. munozi* plots at the interface of mesotheriids, climbers, and fossorial taxa, occupying a distinct but intermediate position due to its combination of moderate brachial indices and exceptionally high wrist MA (MAw = 31.43%; Figure 5c). This position supports the hypothesis that *C. munozi* exhibits a distinct morphofunctional strategy—consistent with wrist‐powered digging—without fully converging on the ecomorphological profiles of extant fossorial mammals. Its position in MFS is primarily structured by BIU and MAe, although its relatively low elbow MA places it near the edge of the mesotheriid cluster. This combination of intermediate brachial proportions and reduced proximal leverage suggests a functionally versatile forelimb, distinct from typical fossorial profiles.

### 3.3 MA for Elbow and Wrist

Results from the MA comparison using the classic elbow model (Figure 1a), the elbow‐plus‐manus model (Figure 1b), and the wrist‐centered model (Figure 1c) are summarized in Table 3 and illustrated in Figure 5.

In the classical elbow model (MAe; Figure 5a), *C*. *munozi* exhibits a relatively low MA value (19.47%) compared to other mesotheriids (mean = 27.9%), placing it closer to climbers (mean = 17.1%) than to fossorial (mean = 31.6%) or terrestrial taxa. Although not the lowest value among mesotheriids, it falls near the lower end of the group's range, suggesting reduced elbow leverage consistent with a less specialized digging strategy.

When incorporating the manus segment into the lever system (MAe + MTCIII‐L; Figure 5b), *C. munozi* shows a further decrease in elbow MA (12.51%), representing a ~7% reduction relative to the classic model. This adjustment places it well within the range of swimmers (mean = 12.8%) and terrestrial mammals (mean = 11.8%), and below that of other mesotheriids (mean = 14.8%). The result supports a functional interpretation of diminished proximal leverage when the manus is engaged.

In the wrist‐centered model (MAw; Figure 5c), *C*. *munozi* exhibits the highest MA recorded among all taxa analyzed (31.43%). This exceeds the upper boundary of the range observed in extant fossorial mammals (mean = 18.4% ± 5.8%; upper bound ~24.2%) by nearly 30% and surpasses the wrist MA of all other mesotheriids (mean = 27.7%). This distinctive value strongly supports the hypothesis of a wrist‐dominated digging strategy, emphasizing distal torque generation as a key component of forelimb function in *C. munozi*.

## 4 Discussion

This study is the first to evaluate forelimb function in *C. munozi* (Notoungulata; Mesotheriidae) through an integrative analysis of functional indices and muscle MA. A long‐standing debate in mesotheriid paleobiology concerns their potential for fossorial behavior (Fernández‐Monescillo et al. 2017; Shockey et al. 2007). Our approach conceptualizes scratch‐digging as a phased sequence of movements involving distal‐to‐proximal kinematic recruitment (Moore et al. 2013).

We evaluated the morphofunctional characteristics of the forelimb in *C. munozi*, and our key findings include: (i) a relatively broad medial epicondyle of the humerus, reflected in elevated values of the Epicondylar Index (EI), suggesting an enlarged surface area for flexor muscle attachment; although other robustness indices (e.g., RRI, URI) do not markedly depart from the mean values observed in mesotheriids or extant terrestrial mammals, the expanded epicondylar morphology stands out as a distinctive osteological feature (Table 3; Figure 4); (ii) an intermediate position in MFS, situated at the interface of mesotheriids, climbers, and fossorial taxa—primarily structured by BIU and MAe, reflecting a combination of intermediate brachial proportions and low elbow MA (Table 3; Figure 3b); and (iii) the highest wrist MA among mesotheriids, supporting a wrist‐powered, distal‐to‐proximal scratch‐digging strategy (Table 3; Figure 5).

This interpretation is reinforced by the distinctive wrist MA, which exceeds that of all extant groups including fossorial mammals. These values suggest that *C. munozi* relied predominantly on wrist flexor torque during the early stages of scratch‐digging, consistent with a distal‐dominated onset of substrate displacement.

### 4.1 *C. munozi* Exhibits Distinct Forelimb Proportions

Although *C. munozi* has been previously described as possessing robust forelimbs (Elissamburu 2012; Shockey et al. 2007), our results reveal a more nuanced scenario. The species presents a relatively broad medial epicondyle and elevated values in both epicondylar and brachial indices (Table 3; Figure 4), yet its overall humeral and radial robustness indices remain moderate compared to *M. cristatum*, which displays the highest values within Mesotheriidae (Figure 3a). This pattern suggests that *C. munozi* did not exhibit maximal diaphyseal thickening, but instead combined modest shaft robustness with expanded distal articular surfaces—potentially reflecting a functional emphasis on torque generation at the elbow and wrist joints, rather than on axial load resistance.

Evidence of ontogenetic variability in limb bone microstructure among mesotheriids (Campos‐Medina et al. 2023) further supports the possibility of adaptive responses to mechanical loading during growth. Interestingly, the holotype of *C. munozi* (SGO.PV.22500) shows smaller diaphyseal dimensions than other specimens attributed to the same species. While this may reflect individual variation, it also raises the possibility that the holotype represents an earlier ontogenetic stage. Given its taxonomic significance, invasive sampling is not currently feasible. However, future application of non‐destructive imaging techniques could help assess its developmental status and refine interpretations of its functional morphology.

Typically, cursorial mammals exhibit high length‐to‐diameter ratios, enhancing stride length and locomotor efficiency (Samuels et al. 2013; Young et al. 2014; Martin et al. 2022). In our osteological PCA (Figure 3a), *C*. *munozi* occupies a region of morphospace overlapping with cursorially‐inclined terrestrial taxa, primarily due to its intermediate values in humeral and radial diameters. Its elevated brachial indices (BIR and BIU) further align with those of cursorial mammals (Figure 4g–h; File S3), suggesting elongation or favorable leverage in proximal limb segments.

However, this cursorial‐like signal contrasts with its position in the functional PCA (Figure 3b), where *C. munozi* plots near the boundary of climbers and fossorial species. This placement is driven by a distinct combination of intermediate BIU and the highest wrist mechanical advantage (MAw) among all taxa analyzed. Notably, *C. munozi* does not fall within the core MFS of climbers or diggers but instead occupies a marginal zone influenced by both traits.

This configuration supports the interpretation of *C. munozi* as a functionally versatile species, combining generalized brachial morphology with specialized distal mechanics. The extreme MAw value may reflect a derived adaptation for wrist‐driven substrate displacement, consistent with a scratch‐digging strategy that does not fully converge on extant fossorial morphotypes.

Nevertheless, *C. munozi* also overlaps in morphospace with fossorial and climbing taxa (Figure 3b), particularly due to its BIU values. These findings suggest functional versatility, aligning with previous assertions that mesotheriids lack the extreme specializations typical of obligate fossorial mammals (Fernández‐Monescillo et al. 2017; Shockey et al. 2007). Instead, the integration of terrestrial, fossorial, and climber taxa features in *C. munozi* supports a facultative fossorial interpretation, whereby digging was likely secondary to terrestrial locomotion.

**Figure 6:**
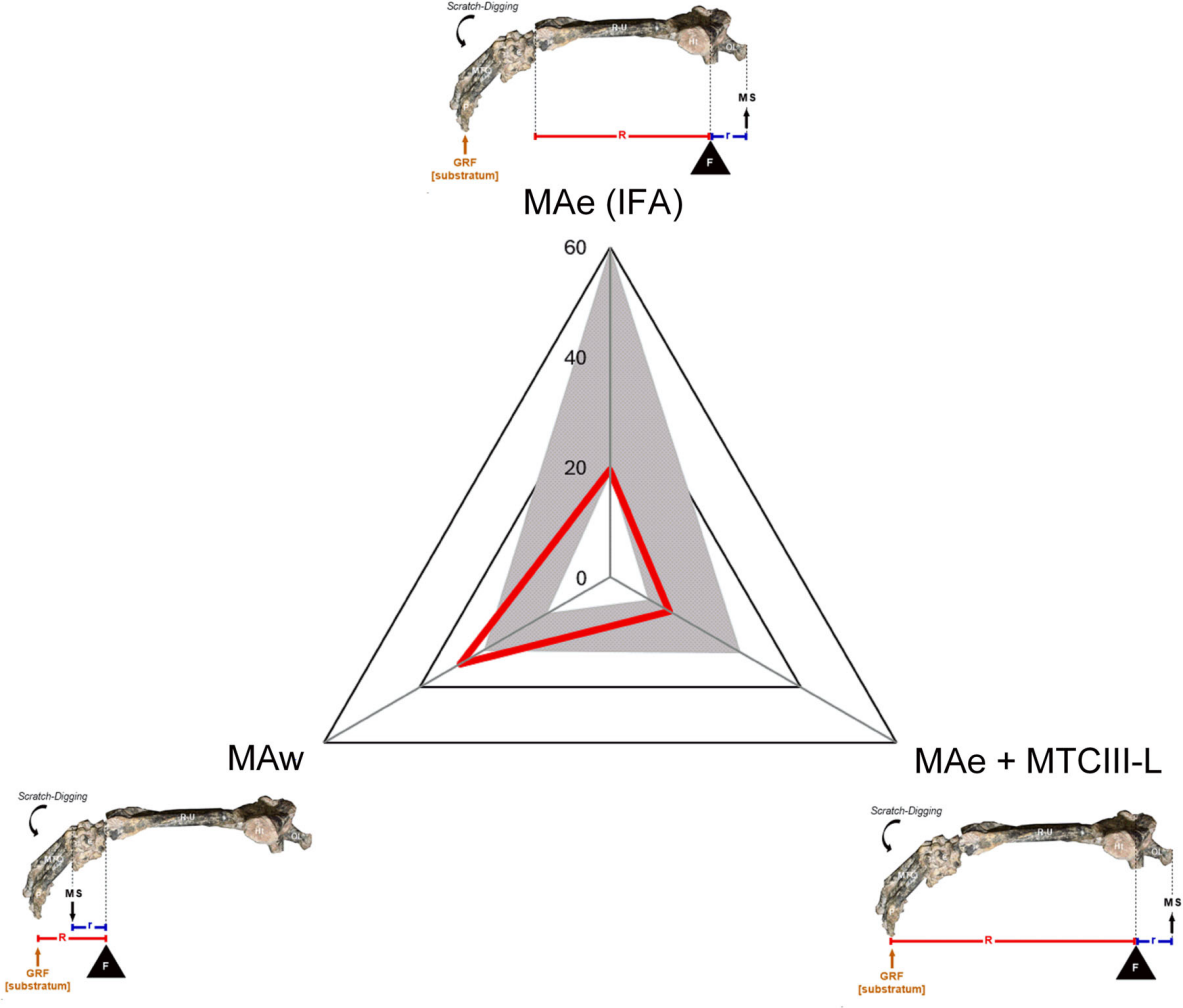
Forelimb morphofunctional space of *Caraguatypotherium munozi* (SGO.PV.22500, MNHN). This radial plot illustrates a conceptual morphofunctional space derived from three mechanical advantage (MA) models representing joint‐level torque potential during scratch‐digging. Each axis corresponds to a distinct biomechanical configuration: Top: Classical elbow mechanical advantage (MAe), equivalent to the Index of Fossorial Ability (IFA); Right: Modified elbow model incorporating the metacarpus and phalanges (MAe + MTCIII‐L); Left: Wrist mechanical advantage (MAw), as defined in this study. Concentric grids represent 20% increments of the maximum recorded values. The shaded gray area delineates the performance envelope of extant scratch‐digging mammals. The red triangle marks the position of *C. munozi*, which exhibits a uniquely high MAw and relatively low MAe. When the manus is included in the elbow model, *C. munozi* shifts closer to the center of the morphofunctional space, supporting an interpretation of wrist‐dominated but integrative excavation mechanics.

These morphospace patterns allow us to evaluate the hypothesis stated in the introduction. *C. munozi* groups with species displaying high wrist MA and reduced elbow leverage—traits commonly associated with scratch‐digging strategies (Moore et al. 2013; Vizcaíno and Milne 2002). The proximity of *C. munozi* to extant taxa that rely heavily on wrist‐based excavation partially supports the hypothesis of morphofunctional similarity. When the manus segment is incorporated into the elbow mechanical model (MAe + MTCIII‐L), the MA of *C. munozi*—initially lower in the classic model—shifts closer to the upper range observed in extant fossorial mammals. This ~7% reduction in MA reconfigures its functional profile toward intermediate locomotor strategies (Table 3; Figure 5b), highlighting the biomechanical relevance of distal segment integration. Such findings reinforce a context‐dependent model in which forelimb segments contribute dynamically to excavation, as visualized in the MFS (Figure 6).

However, not all scratch‐diggers employ the same biomechanical strategy. Some rely more on elbow extension or shoulder retraction depending on muscle architecture, moment arm distribution, and substrate resistance (Moore et al. 2013; Kilbourne and Hutchinson 2019; Elissamburu and De Santis 2011). Moreover, MA use can vary even within a single species, modulated by substrate properties (e.g., density, cohesion) and the animal's internal physiological state. Factors such as fatigue, neuromuscular load distribution, or altered homeostasis may influence joint recruitment and force application.

These considerations highlight the interpretive power of MFSs as structural frameworks for generating hypotheses on how mechanical resources are allocated across joints in response to shifting ecological and physiological constraints. In this sense, our findings align with the Movement Ecology paradigm (Nathan et al. 2008), which emphasizes the dynamic interplay between internal state, motion capacity, navigation capacity, and external environment in shaping movement strategies. Within this framework, *C. munozi* appears to exhibit its greatest mechanical capacity at the wrist, suggesting this joint may have played a predominant role during the initial phases of excavation, particularly when distal torque transmission was biomechanically optimal.

As excavation progressed and fatigue accumulated, *C. munozi* may have shifted toward greater reliance on proximal joints, engaging larger muscle groups with greater aerobic capacity. This pattern aligns with functional models (Kardong 2012) in which MA, muscle architecture, and contraction dynamics interact to determine motor recruitment sequences and task endurance. Such a progression—from wrist to elbow or shoulder—would support a dynamic forelimb strategy, in which excavation begins with efficient distal segments and progressively recruits proximal joints to maintain force output. This multifunctional architecture suggests ecological adaptability, potentially favoring locomotion and substrate interaction across varied terrain types, including sloped or irregular surfaces.

Despite displaying mechanical traits associated with scratch‐digging, particularly elevated wrist MA, *C. munozi* does not cluster with extant fossorial mammals in MFS (Figure 3b). This discrepancy, which persists even after correcting for body size, suggests that mesotheriids evolved forelimb configurations that are functionally distinct from those of modern scratch‐diggers. One possible explanation is the influence of non‐isometric scaling, which can affect both cross‐sectional geometry and lever arm proportions (Biewener 1989; Kilbourne and Hutchinson 2019). The wide range of body sizes in the comparative sample may partially mask ecological signals in PCA‐based analyses. As a result, the position of *C. munozi* near the boundaries of multiple locomotor groups, combined with its decoupled pattern of low elbow leverage and high wrist torque, supports the interpretation of a facultative digging strategy that does not fully converge on the morphotypes of highly specialized fossorial mammals. These findings highlight the importance of integrating osteological evidence with biomechanical modeling to better resolve functional diversity in extinct lineages.

### 4.2 Osteological Indices as Indicators of Muscle Development and Function in *C. munozi*

The osteological signature of *C. munozi* reveals forelimb features indicative of moderate muscular development and functional differentiation within Mesotheriidae. Although this taxon exhibits a relatively broad medial epicondyle (HEB)—a component of the EI (Table 3, Figure 4)—its EI value is intermediate within the clade, lower than those observed in *P. casirense* and *M. cristatum*. This suggests that *C. munozi* did not evolve the most extreme epicondylar expansions within Mesotheriidae, but rather reflects a condition consistent with functional versatility. The moderately enlarged epicondyle likely provided sufficient attachment area for wrist flexors, pronosupinators, and elbow adductors (Elissamburu 2007; Fujiwara and Hutchinson 2012), which contribute to both scratch‐digging and postural stabilization.

In analogous taxa, muscles such as *flexor carpi radialis* and *flexor carpi ulnaris* often exhibit long tendinous insertions that facilitate rapid contraction cycles and torque transmission at the wrist (Sonntag 1922; Macalister 1870). These long tendons, by allowing the muscle belly to remain proximal, can reduce distal limb mass and energetic cost while maintaining effective force output (Alexander and Bennet‐Clark 1977).

The development of elbow adductors may also reflect aspects of posture and limb control. According to Fujiwara and Hutchinson (2012), increased adductor leverage is associated with crouched, semi‐parasagittal limb positions that improve maneuverability and joint stabilization. This is consistent with Biewener (1989) model of posture scaling, where small‐ to medium‐sized mammals tend to adopt crouched gaits for better dynamic control, unlike the erect postures of large‐bodied taxa. Accordingly, the configuration observed in *C. munozi* implies a crouched posture during locomotion and potentially during excavation.

Our PCA of functional indices and MA (Figure 3b) supports this interpretation, placing *C. munozi* at an intermediate position in MFS, near the interface between climbers and fossorial mammals. Its placement is primarily structured by intermediate brachial indices (BIU, BIR) and a relatively high wrist MA. In contrast, other mesotheriids display varied spatial associations: *P. casirense* plots closer to the region occupied by climbers, while *P. achírense* falls within a morphospace shared by fossorial, swimmer, and terrestrial taxa. *T. spegazzinianus* and *M. cristatum* occupy more marginal zones aligned with climber–swimmer gradients. These distinctions suggest functional heterogeneity within Mesotheriidae, with *C. munozi* representing a morphofunctionally versatile form distinct from the extremes represented by its relatives.

### 4.3 Beyond Ratios: Reassessing Functional Indices and MA in the Forelimb of *C. munozi*

Functional indices are widely used in ecomorphological research to infer locomotor strategies, but they do not provide conclusive evidence of ecological function, especially when considered in isolation. Similar index values can result from different anatomical configurations—for example, humeral elongation versus forearm shortening—each with distinct biomechanical implications. Thus, equivalent index scores may reflect convergent limb proportions rather than shared locomotor performance. This limitation is particularly relevant when studying extinct taxa, for which direct behavioral data are lacking. Functional indices should therefore be interpreted with caution and always in conjunction with broader morphofunctional evidence.

Our results illustrate these concerns. Several extant mammals with non‐fossorial habits (e.g., *Bos taurus*, *Sus scrofa*, *Equus ferus caballus*) exhibit high values in the IFA (File S1). By contrast, *C. munozi* displays a relatively low elbow mechanical advantage (MAe = 19.47%) compared with the average for mesotheriids (~27.9%) and fossorial mammals (~31.6%) (Table 3; Figure 5a). When the manus is included in the lever model (MAe + MTCIII‐L), the MA drops further to 12.51%, positioning *C. munozi* closer to swimmers and terrestrial taxa (Figure 5b). This pattern suggests reduced reliance on proximal torque and greater emphasis on distal force production during excavation.

In addition, previous studies have shown that low IFA values may not solely indicate diminished strength but could also be associated with enhanced elbow mobility. A shortened or posteriorly oriented olecranon may allow for increased angular extension, enabling rapid and repetitive forelimb strokes (Vizcaíno et al. 2016). This interpretation aligns with biomechanical findings in glyptodonts, where high IFA values are thought to reflect body support mechanics rather than digging specialization (Vizcaíno et al. 2011). Among fossorial armadillos, IFA variation is similarly interpreted as a trade‐off between speed and force production.

Our data suggest that *C. munozi* may have favored a strategy emphasizing rapid, dynamic distal movements rather than maximum proximal force output. This biomechanical configuration is supported by the combination of low elbow MA, moderate brachial indices, and exceptionally high wrist leverage. Together, these traits outline a forelimb adapted for context‐sensitive excavation, likely optimized for initiating substrate displacement with efficient wrist‐driven torque.

These findings reinforce the idea that functional indices should be viewed as suggestive rather than definitive. While they remain useful proxies for lever mechanics and limb proportions, they must be integrated with biomechanical modeling and ecological context to accurately reconstruct the locomotor adaptations of extinct species.

### 4.4 Wrist MA and Flexor Leverage in *C. munozi*

Our analysis of MA across mesotheriids reveals patterns consistent with classical lever models, particularly the IFA as described by Hildebrand (1985). Incorporating the manus segment into the elbow model (MAe + MTCIII‐L) consistently—and predictably—reduces MA due to increased resistance from substrate reaction forces (Biewener 1989). This adjustment highlights the biomechanical significance of distal segment integration, shifting *C. munozi* toward the statistical range of swimmers and bringing it closer to the lower bound of performance observed in extant diggers, as delimited by their standard error margins (Figure 5b). In this framework, *C. munozi* exhibits elbow MA values comparable to climbers (Figure 5a), yet occupies a distinct region in MFS (Figure 3b). This mismatch underscores the importance of using multiple indices and biomechanical metrics to draw robust functional inferences (Vizcaíno et al. 2016).

As a novel contribution, this study introduces wrist mechanical advantage (MAw; Figure 2d) as a parameter for estimating torque generation around a wrist‐centered rotational axis, accounting for the functional length of metacarpal III. Results show consistently high MAw values across mesotheriids (Figure 5c), with *C. munozi* exhibiting the highest value in the data set (31.43%). This outstanding wrist MA exceeds that of all extant analogs, including specialized scratch‐diggers, and strongly supports a model of excavation driven primarily by distal torque production. Future studies should also consider the biomechanics of manual phalanges, as these elements represent the interface between the individual and the substrate and may play a pivotal role in excavation dynamics.

This pattern is further supported by osteological traits associated with the flexor musculature. While *C. munozi* shows intermediate values for humeral robustness (HRI) relative to other mesotheriids (Table 3; Figure 4a), it presents a relatively broad medial epicondyle and a high EI (Figure 4b), suggesting enlarged insertion areas for wrist flexors, pronators, and elbow adductors (Elissamburu 2007, 2013). These traits are consistent with strong distal motor capabilities. As proposed by Kilbourne and Hoffman (2013), increases in substrate resistance may shift muscle recruitment proximally. In this context, *C. munozi* likely initiated substrate displacement through high‐efficiency wrist flexion, transitioning to elbow and shoulder torque as resistance accumulated.

Taken together, these results suggest a forelimb strategy optimized for initiating scratch‐digging with powerful wrist movements. Further discoveries of the proximal humerus and scapula in *C. munozi* could refine interpretations regarding torque distribution and the complete excavation kinematics of this taxon.

### 4.5 MFSs for Scratch‐Digging in *C. munozi*

To clarify how scratch‐digging was biomechanically executed in *C. munozi*, we constructed an MFS based on three MA models: the classical elbow model (MAe), an elbow model incorporating manus length (MAe + MTCIII‐L), and a novel wrist‐centered model (MAw) (Figure 5). The classical elbow model positions *C. munozi* near the lower edge of the fossorial range, whereas its MAw exceeds all extant analogs—including specialized diggers—by approximately 9%, suggesting a shift in emphasis toward distal torque generation rather than proximal leverage.

Incorporating the manus segment into the elbow model further reduces MA by ~7% compared to the classical configuration. This drop reflects the functional cost of distal integration: Increasing the lever arm length exposes the joint to greater substrate resistance (Biewener 1989), thereby reducing effective elbow torque. Yet, despite this decline, the adjusted MAe + MTCIII‐L value still falls within the lower threshold of extant fossorial mammals (as delimited by their standard error), indicating that *C. munozi* may have retained functional consistency with scratch‐digging strategies, though through an alternative mechanical configuration.

These results challenge the widespread reliance on OL—and thus IFA—as a universal predictor of fossorial behavior (Hildebrand 1985; Elissamburu and De Santis 2011). In *C. munozi*, a relatively low IFA coincides with anatomical signals of limited elbow excursion, such as a modest olecranon process. However, the high MAw values suggest a compensatory mechanism in which increased wrist leverage offsets diminished elbow performance. This supports the hypothesis that wrist‐powered excavation may represent a distinct functional pathway in fossorial evolution, particularly in mammals with intermediate brachial proportions.

The triangular radial MFS (Figure 6) synthesizes this model‐based evidence. When the three MA values are visualized together, *C. munozi* occupies a central yet asymmetric position—anchored by its exceptional wrist leverage and balanced by reduced elbow torque when distal segments are incorporated. This configuration emphasizes the importance of evaluating complete forelimb mechanics, from the shoulder to the contact point with the substrate. Analyses limited to proximal segments may overlook critical distal contributions, especially in extinct taxa where functional inferences depend heavily on morphological integration and biomechanical modeling.

Altogether, the integration of MAe, MAe + MTCIII‐L, and MAw illustrates the biomechanical distinctiveness of *C. munozi* within the context of scratch‐digging mammals. Rather than maximizing elbow torque, this taxon achieves high functional output via the wrist, supporting a digging strategy that begins distally and progressively recruits proximal joints. This composite pattern reinforces the importance of incorporating the entire limb—including the manus and terminal phalanges—into functional reconstructions. Such comprehensive models are essential not only for resolving alternative excavation strategies in extinct mammals, but also for advancing our understanding of forelimb evolution and morphofunctional diversity across terrestrial vertebrates.

### 4.6 Limitations and Future Directions

To further refine the MFS developed in this study, future research should integrate MA estimates with angular excursion data across key forelimb joints. While our current model is grounded in osteological proxies of static leverage at the elbow and wrist, a more complete understanding of motor capacity would benefit from incorporating joint mobility profiles tied to functional output. Bridging structural indicators of force generation (e.g., moment arms of triceps and wrist flexors) with joint excursion patterns during scratch‐digging behaviors would enable more dynamic assessments of locomotor performance. Such integration aligns with a broader framework of biological function and capacity, where structure and performance are co‐analyzed rather than inferred independently.

In addition, future work should expand comparative kinematic datasets across extant mammals with diverse locomotor strategies and forelimb anatomies. This would enhance analog‐based inferences in fossil taxa such as mesotheriids, especially in wrist‐dominant excavators. A broader sampling of scratch‐digging behaviors—beyond traditionally studied diggers—could refine our understanding of loading regimes and motor strategies during excavation.

The development of MFSs would also benefit from the integration of mechanobiological data, particularly bone microstructural adaptations to mechanical loading (Cutini et al. 2014; Lieberman et al. 2004). High‐resolution imaging (e.g., Brassey and Sellers 2014) and finite element simulations (Rayfield 2007) can further clarify stress distributions and mechanical optimization within joints. Time‐series datasets of joint kinematics in extant species (Catavitello et al. 2018) may help characterize lineage‐specific solutions and increase the dimensionality of functional spaces, ultimately improving the reliability of paleobiological inferences.

Although limited to a single extinct species, the findings presented here address broader questions about the evolutionary dynamics and functional constraints of mammalian excavation. In this context, constraints refer to biomechanical limitations—such as joint shape, lever geometry, or muscle attachment distribution—that restrict the set of viable anatomical solutions for producing force in scratch‐digging systems (Losos 2011). These constraints may channel evolutionary outcomes toward specific morphotypes, even under divergent ecological pressures. The case of *C. munozi*, which exhibits increased MA at the wrist and relatively limited elbow torque, illustrates how anatomically analogous solutions can emerge across distantly related clades—such as xenarthrans, caviomorph rodents, and marsupials—facing similar biomechanical demands (Elissamburu and De Santis 2011; Moore et al. 2013).

Moreover, while *C. munozi* does not fully converge on the morphospace of extant fossorial taxa, its unique forelimb configuration provides evidence of morphofunctional similarity shaped by distal leverage. These patterns reinforce the idea that certain limb architectures may represent biomechanically optimal solutions within constrained functional envelopes (Biewener 1989; Carrano 1997). This supports the broader concept of evolutionary “design filters” in the shaping of form–function relationships.

Importantly, our data also raise questions about the ontogenetic status of the holotype of *C. munozi* (SGO.PV.22500). Although it displays relatively small diaphyseal dimensions compared to other individuals attributed to the same species (Campos‐Medina et al. 2023), it remains unclear whether this reflects individual variation or an earlier developmental stage. Given its status as the holotype, histological sampling is not advisable. However, future studies using noninvasive imaging techniques—such as high‐resolution CT or synchrotron scanning—may clarify its growth stage and improve interpretations of limb robustness and functional capacity.

Finally, the distinct morphofunctional signature of *C. munozi* offers a framework for interpreting larger‐scale patterns in mammalian forelimb evolution. In arid, tectonically dynamic Miocene environments—especially those at intermediate latitudes such as the southern Central Andes—behavioral adaptation was likely shaped by a combination of substrate disruption, climatic variability, and localized ecological pressures (Flynn et al. 2005; Gomes Rodrigues et al. 2018). In this context, forelimb morphology may have reflected not only biomechanical function but also thermoregulatory demands (Allen 1877; Lovegrove 2004). The power‐focused architecture of *C. munozi*, particularly its exceptionally high wrist MA, may represent a morphofunctional innovation shaped by such environmental constraints. Its unique placement within MFS underscores the role of ecological filtering in generating biomechanical diversity. This case highlights the utility of integrative approaches for reconstructing niche dynamics and locomotor strategies in extinct mammals, particularly within heterogeneous and transitional Miocene landscapes.

## 5 Conclusion

The MFS framework developed in this study offers a refined perspective on the long‐standing hypothesis of fossoriality in Mesotheriidae. Using the forelimb of *C. munozi* (SGO.PV.22500, MNHN) as a model, we quantified MA at the elbow and wrist and integrated these measures into a multivariate framework to evaluate locomotor strategies. This approach allowed us to move beyond categorical classifications and identify a biomechanical configuration consistent with wrist‐driven scratch‐digging.

Our findings reveal that mesotheriids, particularly *C. munozi*, possess a forelimb structure characterized by elevated wrist MA relative to the elbow. In *C. munozi*, wrist MA reaches 31.43%—the highest among all sampled taxa—suggesting a predominant role for wrist torque in initiating excavation. This pattern supports a distal‐to‐proximal recruitment sequence, in which the wrist initiates substrate displacement, followed by the engagement of more proximal joints as resistance increases. Such a strategy aligns with current models of joint function in digging mammals and highlights a distinct mechanical solution within Mesotheriidae.

This configuration challenges prior interpretations that portrayed mesotheriids as uniformly fossorial or broadly generalized diggers (e.g., Shockey et al. 2007; Fernández‐Monescillo et al. 2017). By combining osteological indices with MA metrics, our analysis reveals functional diversity within the clade. Rather than fitting a single excavatory archetype, mesotheriids appear to occupy a spectrum of morphofunctional strategies shaped by lineage‐specific adaptations and ecological demands. *C. munozi* emerges as a specialized form, relying heavily on distal leverage rather than proximal force generation.

While our results emphasize the role of the wrist in *C. munozi*, we caution against overinterpreting individual indices such as elbow MA (IFA) without broader taxonomic sampling. Some non‐fossorial mammals in our data set exhibit high IFA values, suggesting that this index alone is not a reliable predictor of digging behavior. Expanding the sample to include more diverse fossorial mammals—especially large‐bodied and highly specialized diggers—will be crucial for testing the generality of these findings. Future research should also integrate kinematic data, joint excursion ranges, and bone microstructural adaptations to better contextualize static biomechanical proxies.

The distinct placement of *C. munozi* in morphospace may also reflect ecological filtering. This taxon inhabited a tectonically active, arid Miocene environment at intermediate latitudes, where substrate variability, climatic stress, and resource dynamics could have selected for compact, torque‐efficient limbs (Flynn et al. 2005; Gomes Rodrigues et al. 2018). The combination of elevated wrist MA, moderate brachial proportions, and an intermediate morphospace position suggests a morphofunctional innovation influenced by both biomechanical and environmental constraints.

In sum, this study illustrates the value of integrating MA and osteological indices within a multivariate framework to reconstruct locomotor strategies in extinct mammals. The case of *C. munozi* highlights the evolutionary significance of distal specialization in excavation and underscores the importance of comprehensive, functionally grounded models for understanding the diversity of mammalian limb evolution.

## Author Contributions

**Paul Medina‐González:** conceptualization, investigation, funding acquisition, writing – original draft, methodology, validation, visualization, writing – review and editing, software, formal analysis, project administration, data curation, resources. **Karen Moreno:** conceptualization, writing – original draft, writing – review and editing.

## Ethics Statement

Fossil specimens of *Caraguatypotherium munozi* were accessed and examined under official authorization granted by the Chilean National Monuments Council (Consejo de Monumentos Nacionales, CMN), via letter No. 1355/2023 (CMN‐PNP N° 102‐2023), within the framework of FONDECYT project No. 11231111.

## Conflicts of Interest

The authors declare no conflicts of interest.

## Peer Review

The peer review history for this article is available at https://www.webofscience.com/api/gateway/wos/peer-review/10.1002/jmor.70069.

## Supporting information

Supporting file 1.

Supporting file 2.

Supporting file 3.

## Data Availability

All data, measurements, indices, and analysis scripts used in this study are included in the main text and supporting information. Upon acceptance, datasets will be deposited in Zenodo and cited in the final version of the manuscript. All data and R scripts used for MA and PCA analyses are available through Zenodo (DOI: 10.5281/zenodo.15189182). The record is publicly accessible, but files are currently under restricted access for peer review purposes. Files can be shared upon request with the editor or reviewers and will be made fully public upon acceptance of the manuscript.
